# A Rare Collision Medullary and Papillary Thyroid Carcinoma in Autoimmune Thyroid Disease: Case Report

**DOI:** 10.32604/or.2025.072100

**Published:** 2026-02-24

**Authors:** Tena Šimunjak, Bernardica Jurić, Andro Košec, Vladimir Bedeković

**Affiliations:** 1 Department of Otorhinolaryngology & Head and Neck Surgery, University Hospital Sveti Duh, Zagreb, 10000, Croatia; 2 Ljudevit Jurak Clinical Department of Pathology and Cytology, University Clinical Hospital Center Sestre Milosrdnice, Zagreb, 10000, Croatia; 3 Department of Otorhinolaryngology & Head and Neck Surgery, University Hospital Center Sestre Milosrdnice, Zagreb, 10000, Croatia; 4 School of Medicine, University of Zagreb, Zagreb, 10000, Croatia

**Keywords:** Medullary thyroid carcinoma, papillary thyroid carcinoma, collision tumor, calcitonin, graves’, disease, case report

## Abstract

Background: Collision medullary and papillary thyroid carcinoma (MTC/PTC) is a rare entity, constituting less than 1% of all thyroid malignancies. The concurrent presence of these malignancies in patients with autoimmune thyroid disease, such as Graves’ disease, is even more uncommon. Calcitonin (Ctn) is considered one of the key MTC biomarkers. Mixed tumors may alter this relationship. Case Description: We report the case of a 55-year-old female with a history of Graves’ disease, who underwent total thyroidectomy for persistent dysthyroid orbitopathy. Histopathological analysis revealed a 9-mm collision MTC/PTC tumor in the left thyroid lobe, confirmed through immunohistochemical staining. Postoperative evaluation demonstrated lymph node metastases, necessitating central and left lateral neck dissection. Postoperative serum markers (calcitonin, carcinoembryonic antigen, thyroglobulin) declined significantly following surgery and radioiodine therapy. Conclusion: Subcentimeter collision MTC–PTC tumors can be aggressive, challenging size-based management thresholds. Treatment should integrate MTC and PTC protocols, with Ctn, carcinoembryonic antigen (CEA), and thyroglobulin monitored in tandem. Larger datasets are needed to refine Ctn prognostic thresholds in mixed tumors.

## 1 Introduction

The simultaneous occurrence of mixed medullary thyroid carcinoma (MTC) and papillary thyroid carcinoma (PTC) within the same thyroid gland is an exceptionally uncommon phenomenon, accounting for <1% of all thyroid carcinomas [1]. Even more infrequent is its occurrence in patients with a background of autoimmune thyroid disorders, such as Graves’ disease. In this report, we present a rare case of collision-type MTC and PTC with a focus on evaluation, tumor behavior, and diagnostic and therapeutic considerations, given the small size of the lesion and its aggressive course.

This study was waived approval by the ethics committee of the Ethical Committee of the University Clinical Hospital Center Sestre Milosrdnice due to minimal risk to the privacy of the patient and the fact that the research could not practicably be conducted without the waiver according to ICMJE guidelines. Written informed consent was obtained from the patient, and the study was prepared according to the CARE case report guidelines, and a CARE checklist was provided [2]. Please see Supplementary Material S1 for more details.

### Case Presentation

A 55-year-old female patient, with a history of a null cell pituitary adenoma (underwent surgery 8 years prior), was diagnosed with Graves’ disease in the Department of Endocrine Disease, University Hospital Center Sestre milosrdnice in Zagreb, Croatia. The patient had been on thiamazole for 7 years, with regular ultrasound (US) monitoring every two years, with the last US showing inhomogeneous glandular structure and normal dimensions. Her general health had been unremarkable otherwise, and the hormonal status after null cell pituitary adenoma treatment was within reference levels, since the tumor had been confirmed as hormonally inactive prior to surgery and during follow-up. Due to poorly regulated thyroid hormonal status and the presence of dysthyroid orbitopathy, the patient was counseled to proceed with surgical treatment, and a total thyroidectomy was performed.

**Figure 1:**
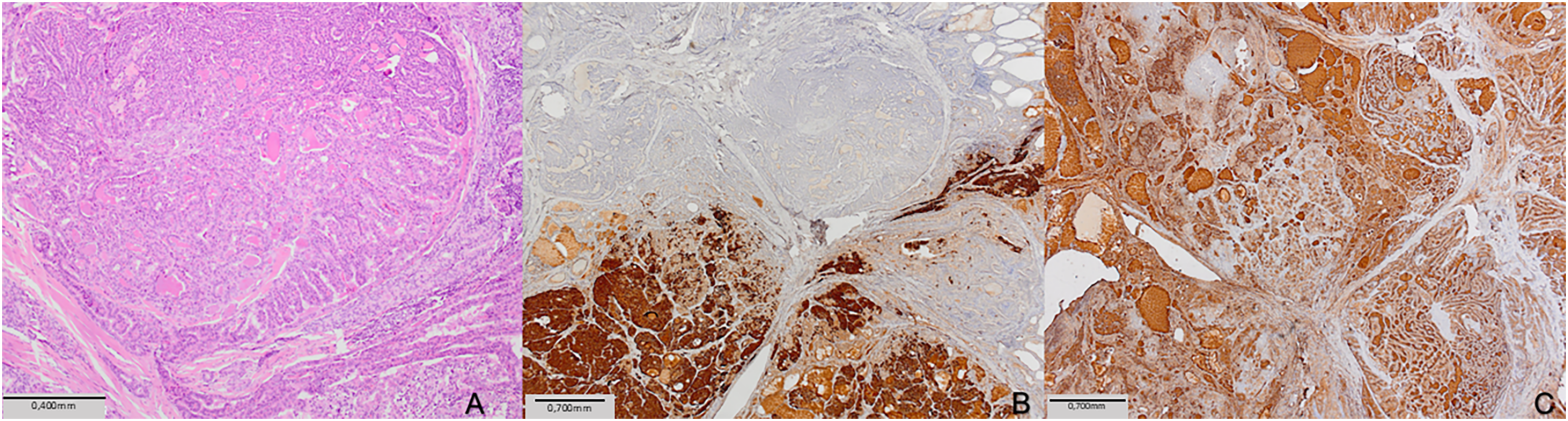
Histopathological examination of the left lobe thyroid mass: (**A**) Tumor was a mixture of two components (HE, ×100; scale bar = 0.4 mm). (**B**) Immunostaining for calcitonin showed positive reaction in medullary carcinoma component, and negative in papillary carcinoma component (calcitonin, ×40; scale bar = 0.7 mm). (**C**) Immunostaining for thyroglobulin showedpositive reaction in papillary carcinoma component, and negative in medullary carcinoma component (thyroglobulin, ×40; scale bar = 0.7 mm)

**Figure 2:**
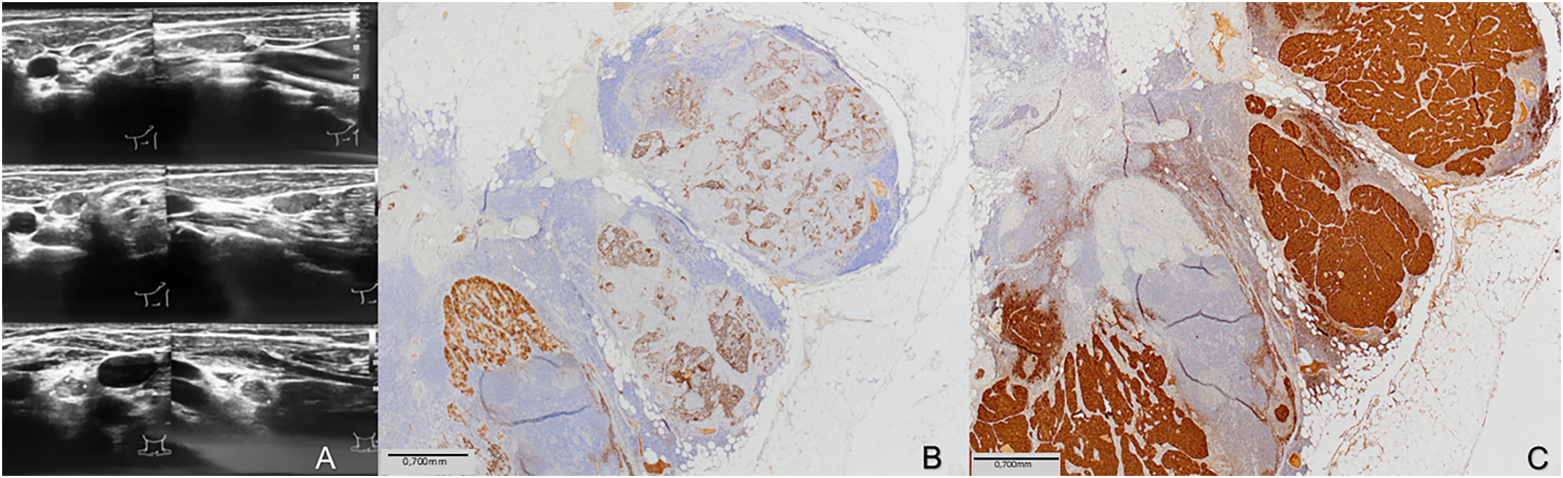
Imaging and histopathological correlation of metastatic lymph nodes in the left neck. (**A**) Ultrasound images demonstrate pathologic lymph nodes in the left neck with features consistent with malignancy, including round shape, hypoechoic texture, and absence of hilum. (**B**) Histological section of the excised lymph node with immunohistochemical staining for thyroglobulin, showing positive staining in the metastatic papillary thyroid carcinoma component (thyroglobulin, ×40; scale bar = 0.7 mm). (**C**) Adjacent section stained for calcitonin, highlighting the presence of medullary thyroid carcinoma cells within the same lymph node (calcitonin, ×40; scale bar = 0.7 mm)

On histology, a 9 mm solid nodule was identified in the left lobe of the thyroid gland. The right lobe was unremarkable. Histopathologically, the tumor consisted of papillary fronds with a fibrovascular core, but also composed of a dispersed cell population of round plasmacytoid cells with moderate granular cytoplasm. The presence of dual tumor populations was confirmed by immunohistochemistry staining. Tumor cells were positive for calcitonin and negative for thyroglobulin in one component, while they were negative for calcitonin and positive for thyroglobulin in the other component (Fig. 1). The following was, by the fifth revision of the WHO classification of thyroid tumors, enough to establish the diagnosis of mixed medullary and follicular cell-derived thyroid carcinoma. Due to this histopathologic diagnosis, additional postoperative workup was performed. Levels of carcinoembryonic antigen (CEA) tumor marker were elevated (31 μg/L), and head and neck US described suspicious nodal formations, one on the left paratracheal side (6 mm × 7 mm × 9 mm), with a second found in the III/IV left neck region (7 mm × 10 mm × 16 mm) and a third, more caudal, identified in the left IV neck region (7 mm × 9 mm × 12 mm) (Fig. 2). Fine needle aspiration (FNA) found elevated levels of both calcitonin (Ct) and thyreoglobulin (Tg) (Table 1). As findings were microscopically suggestive of metastasis of mixed MTC and PTC, central compartment and left lateral neck dissection (levels II–V) were performed. Hystopatology revealed metastases of mixed medullary and papillary thyroid carcinoma, of the collision subtype isolated in six lateral lymph nodes and in five lymph nodes in the central compartment. Postoperative Tg, Ct, and CEA levels were reduced (Ct: 6.3 ng/L, Tg < 0.09 μg/L; CEA: 4.6 μg/L) and the patient was administered 104 mCi of radioiodine (RAI) therapy. One year after treatment, the patient is still disease-free, and is planned to continue US follow-up every two months, followed by Tg, Ct and CEA blood level testing. Although she remains under frequent follow-up, careful interpretation of disease-free status is required due to short follow-up.

**Table 1:** Concentrations of calcitonin (Ct) and thyroglobulin (Tg) in fine-needle aspiration (FNA) samples from metastatic lymph nodes in the left neck

Neck region	Calcitonin (ng/L)	Thyreoglobulin (μg/L)
**III**	4938.1 ng/L	83.3 μg/L
**IV**	1052.2 ng/L	1421.84 μg/L
**VI**	1257.2 ng/L	3550.96 μg/L

## 2 Discussion

The simultaneous presence of MTC and PTC within the thyroid gland is exceedingly rare, and no data on incidence is known, since the cases are limited to small series over several decades. MTC originates from parafollicular C cells, can occur either sporadically (in 75% of cases) or as part of hereditary syndromes such as multiple endocrine neoplasia types 2A and 2B (MEN2A and MEN2B), both of which are associated with mutations in the RET proto-oncogene. In contrast, PTC arises from follicular cells and is associated with BRAF or RAS mutations [2]. The literature describes these collision tumors as having complex etiology and behavior, often attributed to potential pathways such as a common stem cell origin, transformation of medullary cells into follicular-type cells, or independent tumor formation within the same gland [3]. Ciampi et al. [4] evaluated 24 patients with concurrent MTC and PTC for RET, BRAF, and RAS mutations. The two tumor types generally carried distinct alterations; RET or RAS mutations predominated in MTC, while some PTCs harbored BRAF^V600E^ or RAS changes, and many had no detectable mutation. No identical point mutation was present in both tumor types from the same patient, supporting the hypothesis that these malignancies arise independently rather than from a single progenitor clone. These rare tumors are reported to appear as: 1) distinct or independent tumors (MTC and PTC appearing separately in different locations of the gland); 2) synchronous tumors occurring in separate anatomical lobes, 3) true mixed tumors (a single tumor exhibiting both MTC and PTC cellular features), and 4) collision tumors [1,5]. Unfortunately, due to budget constraints, genetic testing was not carried out in our patient, and represents a significant limitation in the era of precision medicine. Adding this information might have provided for an additional hypothesis on etiopathogenesis of collision tumor formation and improved on the current level of knowledge.

Our case represents a collision-type tumor, the second rarest presentation, where MTC and PTC coexisted in an anatomically discrete but abutting arrangement. In published literature, the size of reported collision MTC/PTC tumors is not always provided, especially regarding subtypes. In our patient, the tumor (MTC + PTC) was small (9 mm in diameter) but displayed an aggressive course with lymph node metastasis. Generally, a 9 mm thyroid nodule is below the size threshold where guidelines regularly suggest fine-needle aspiration biopsy (FNAB) as necessary, as small lesions are often considered less aggressive [6,7]. However, our case underscores that even small collision tumors may warrant aggressive management. Typically, preoperative Ct levels in MTC correlate closely with tumor size, nodal disease, and distant spread, and can therefore inform decisions about the extent of surgery, although its role remains debated. The American Thyroid Association (ATA) guidelines suggest that in the absence of ultrasound-detected cervical metastases or distant disease, consideration may be given to performing a lateral neck dissection (levels II–V) based on serum calcitonin levels [8]. Nevertheless, studies suggest that in mixed MTC/PTC cases, this correlation is less pronounced, likely due to the influence of the PTC component [5]. Indeed, one study noted that preoperative Ct levels in synchronous MTC/PTC lesions did not correlate with lymph node metastasis, in contrast to simple MTC, where Ct levels serve as a reliable predictor [1,7]. We found elevated calcitonin and thyroglobulin in a suspect lymph node in our case, prompting further surgical treatment, but in these tumors, it should be noted that the absence of calcitonin elevation cannot rule out malignancy. In Table 2, we summarized case reports and series illustrating these patterns in cases of mixed MTC/PTC cases.

**Table 2:** Summary of cases of mixed medullary (MTC) and papillary thyroid carcinoma (PTC) cases, showing preoperative calcitonin (Ctn, pg/mL; converted values noted), tumor size, cervical nodal status, and metastatic composition (MTC only, PTC only, or both). “N/A” = data not reported; tumor size = largest dimension of each component

Author (Year)	Ctn preoperative level	Tumor size	Cervical metastasis	Metastatic component
Gero et al. (1989) [9]	N/A	N/A	N/A	N/A
Albores-Saavedra et al. (1990) [10]	N/A	N/A	Yes	Both MTC & PTC
Apel et al. (1994) [11]	413 pg/mL (converted from ng/L)	84 g tumor mass	Yes	Both MTC & PTC
Lax et al. (1994) Case 1 [12]	Elevated (not quantified)	5 cm	Yes	Both MTC & PTC
Lax et al. (1994) Case 2 [12]	Elevated (not quantified)	N/A	Yes	Both MTC & PTC
Lax et al. (1994) Case 3 [12]	Elevated (not quantified)	2.6 cm	Yes	Both MTC & PTC
Shiroko et al. (2001) [13]	1,600,000 pg/mL (converted from ng/mL)	1.8 cm × 1.8 cm PTC and MTC	Yes	Both MTC & PTC
Merchant et al. (2002) [14]	6.295 pg/mL	1.5 cm MTC and a 0.3 cm PTC	No	–
Seki et al. (2004) Case 1 [15]	32.6 pg/mL	6.5 cm × 5.0 cm × 3.5 cm MTC and 0.7 cm PTC	Yes	Both MTC & PTC
Seki et al. (2004) Case 2 [15]	1600 pg/mL	6.0 cm × 4.3 cm × 2.3 cm MTC and 0.3 cm PTC	Yes	Both MTC & PTC
Younes et al. (2005) [16]	767 pg/mL	2 cm × 1 cm PTC and MTC	Yes	MTC only
Rossi et al. (2005) Case 1 [17]	894 pg/mL	2.8 cm MTC, 0.4 cm PTC and 0.6 cm PTC	Yes	PTC only
Rossi et al. (2005) Case 2 [17]	N/A	1.1 cm PTC and MTC	No	–
Rossi et al. (2005) Case 3 [17]	Normal serum Ctn	1.1 cm PTC and MTC	No	–
Dionigi et al. (2007) Case 1 [18]	Elevated serum 294 pg/mL	4 cm MTC + 0.4 cm PTC	Yes	Both MTC & PTC
Dionigi et al. (2007) Case 2 [18]	1000 pg/mL	2 cm MTC + 0.3 cm PTC tall cell	Yes	Both MTC & PTC
Nangue et al. (2009) [19]	1140 pg/mL (converted from ng/L)	3 cm MTC + 0.6 cm PTC	Yes	MTC only
Gurkan et al. (2014) Case 1 [20]	700 pg/mL	3.7 cm and 1.5 cm MTC and PTC	Yes	Both MTC & PTC
Gurkan et al. (2014) Case 2 [20]	750 pg/mL	10 cm MTC and PTC	Yes	Both MTC & PTC
Guerreiro et al. (2021) [21]	428.5 pg/mL	23 mm MTC and PTC	Yes	Both MTC & PTC
Thomas et al. (2021)—21 case from 2009 to 2019) [22]	Elevated serum mean value: 9115 pg/mL (range from 568 to 13,731 pg/mL)	Mean MTC size 3.12 cm; Mean PTC size 0.91 cm	Yes 16/21	14 MTC only 2 PTC only 2 Both MTC & PTC
Zhang et al. (2023)—30 cases retrospective [1]	Elevated serum mean value 133.7 ± 196.4 pg/mL.	Mean MTC size 1.6 ± 2.0 cm; Mean PTC size 0.9 ± 1.9 cm	Yes 15/30	9 PTC only 4 MTC only 2 Both MTC & PTC
Yang et al. (2024) [23]	Elevated not quantified	4.5 cm MTC and PTC	Yes	Both MTC & PTC

Note: MTC, medullary thyroid carcinoma; PTC, papillary thyroid carcinoma; Ct, calcitonin; Tg, thyroglobulin; Units: pg/mL (picograms per milliliter), μg/L (micrograms per liter); “–” indicates a value not reported or not available.

All individual data in this table are extracted from previously published case reports and case series (cited in the table). The pooled descriptive median and interquartile range were calculated by the authors based on these published values from Table 2 to facilitate comparison; Quantifiable preoperative calcitonin values were generally high (median ~760 pg/mL; interquartile range (IQR) ~420–1105 pg/mL), and most node-positive patients had levels well above 400–500 pg/mL. While the dataset is small and imbalanced (only one numeric node-negative case), these findings align with published thresholds for MTC that associate rising basal calcitonin (>20–50 pg/mL) with lateral neck metastases and higher values (>200 pg/mL) with greater lateral/contralateral risk; levels around ≥500 pg/mL have been linked to distant metastasis in several series [8]. Interestingly, we can see the two largest series showed strikingly different median calcitonin levels, Guerreiro et al. [22] reported ~9115 pg/mL with a 76% metastasis rate, whereas Zhang et al. [1] reported ~134 pg/mL with a 50% metastasis rate likely reflecting differences in MTC tumor burden, histologic predominance, case selection, and assay methodology. Where metastatic histology was reported, calcitonin levels were similarly elevated whether nodes contained only MTC or both MTC and PTC, suggesting that systemic calcitonin reflects overall MTC activity irrespective of metastatic composition. Diagnosing medullary thyroid carcinoma (MTC) remains challenging because its cytologic and sonographic features often overlap with other thyroid lesions. On FNA, MTC can closely resemble primary or metastatic tumors, with oncocytic and follicular variants particularly vulnerable to misclassification. Sonographically, it lacks the distinctive suspicious patterns seen in papillary thyroid carcinoma (PTC), such as a taller-than-wide shape or microcalcifications, which may lead to under-recognition [24]. Hence, in mixed pathology, it can be expected that a thyroid lesion may not always display clear and typical ultrasonographic features. These diagnostic blind spots underscore the value of adjunctive testing. Evidence from a meta-analysis by Liu et al. [25] shows that relying on FNA cytology alone yields inconsistent sensitivity (12%–88%, mean ~56%), whereas adding calcitonin washout from the needle rinse raises sensitivity above 95%. Molecular tests, such as the Afirma MTC classifier, ThyGeNEXT with ThyraMIR, and ThyroSeq panels with RET analysis, have also demonstrated high sensitivity for MTC detection. The extent of surgical resection in collision MTC/PTC tumours remains controversial. Revised ATA guidelines for medullary thyroid carcinoma advocate total thyroidectomy with central (level VI) neck dissection in all patients and recommend ipsilateral lateral neck dissection when the basal calcitonin level is ≥20–50 pg/mL; contralateral dissection can be considered when calcitonin exceeds 200 pg/mL [26]. Skip metastasis has also been described in papillary carcinoma, occurring in small upper-pole tumors and older patients, supporting vigilance in evaluating the lateral compartment even when central nodes appear uninvolved [27]. In our case, both sonography and FNA washouts identified nodal metastases. Consequently, we performed central and left-lateral neck dissection (levels II–V). This approach allowed removal of metastatic nodes while sparing the right side, which showed no radiologic or biochemical evidence of disease. Interestingly, our patient’s history of Graves’ disease adds a further layer of complexity. Graves’ disease is more commonly associated with PTC than with MTC [28], and the occurrence of PTC in mixed MTC/PTC tumors has been linked to a higher incidence of thyroiditis [6]. This association raises questions about whether autoimmune thyroid disorders might play a role in the pathogenesis of mixed thyroid tumors. We can speculate that the inflammatory environment associated with autoimmune conditions may contribute to the development or progression of these malignancies, although this relationship remains under investigation.

The coexistence of Graves’ disease and thyroid carcinoma leads to an increased risk of developing malignancy compared to patients without autoimmune thyroid disease. Clinically, the prognosis of patients with combined disease is often unfavorable, showing high probability of malignancy, increased risk of relapse, elevated mortality rates, and high rates of metastasis. Literature suggests several molecular and immunological aspects that link the autoimmune microenvironment to tumor development and progression, where chronic inflammation caused by autoimmune thyroid disease is considered a potential factor that increases the risk of developing thyroid cancer. This may occur through oxidative DNA damage, with upregulation of expression of 8-hydroxy-2-deoxyguanosine (8-OHdG) due to oxidative stress related to autoimmune disease, or microRNA dysregulation (IHC/Serological Implication). MicroRNAs (miRNAs) are key negative gene regulators involved in cellular functions like proliferation and differentiation. Analysis of PTC-related miRNAs showed that patients with autoimmune thyroid disease presented intermediate levels of expression for miRNAs 146b, 221, and 222 when compared with normal thyroid tissue and papillary thyroid carcinoma tissues [29]. The immune response observed in autoimmune thyroid disease is highly aggressive, resulting in cell destruction, whereas the immune response triggered against thyroid cancer is often more tolerant, allowing tumor growth. However, the specific inflammatory infiltrate in Graves’ disease–associated thyroid carcinoma (GD/TC) presents mixed signals regarding tumor aggressiveness. When the inflammatory infiltrate of patients with TC and GD was analyzed, researchers observed a high concentration of activated NK cells and a higher ratio of M1/M2 macrophages. These factors are thought to provide a more effective form of tumor immunity, possibly contributing to the low aggressiveness of TC. This protective profile of NK cells and macrophages (M1/M2 ratio) is cited as having an “indifferent” effect on overall prognosis in combined thyroid disease [29]. Serological markers such as thyroid-stimulating hormone (TSH) receptor antibodies (TRAb) and other autoantibodies play a crucial role in the diagnosis, prognosis, and management of autoimmune thyroid diseases and thyroid cancer. TRAb are indicative of disease activity and can help differentiate it from Hashimoto’s thyroiditis, where anti-thyroid peroxidase (TPOAb) and anti-thyroglobulin antibodies (TgAb) are more prevalent. In the context of thyroid cancer, particularly differentiated thyroid carcinomas such as papillary and follicular types, serological markers like elevated serum thyroglobulin (Tg) are essential for postoperative surveillance and detection of recurrence. The presence of anti-thyroglobulin antibodies can complicate these assessments by interfering with Tg measurement, emphasizing the importance of understanding autoantibody profiles. Functional studies, including assays of receptor activity and cellular responses, complement serological markers by elucidating the pathogenic mechanisms underlying autoimmune processes and neoplastic transformation. For example, evaluating TSH receptor signaling activity can offer insights into the autoimmune stimulation in Graves’ disease and its potential impact on thyroid tumorigenesis [29].

The management of mixed tumors is challenging due to the distinct therapeutic approaches required for each component. For endocrinologists and surgeons, encountering a dual MTC/PTC case requires applying management principles for both malignancies. For MTC, this includes preoperative RET mutation testing, evaluation for associated conditions such as pheochromocytoma in the context of MEN2, and planning for surgery with total thyroidectomy and central neck dissection. Postoperatively, monitoring of calcitonin (Ct) and carcinoembryonic antigen (CEA) levels is essential. As mentioned earlier, the ATA guidelines indicate that prophylactic neck dissection may be guided by preoperative basal calcitonin concentrations [26]. In patients with locally advanced or metastatic RET-mutated MTC, neoadjuvant therapy with RET-targeted kinase inhibitors is an emerging strategy. A small series reported that four patients treated preoperatively with selpercatinib for 4–6 months achieved improved locoregional disease control [30]. For unresectable or metastatic disease, systemic therapy is reserved for progressive or symptomatic cases not suitable for local intervention. Selective RET inhibitors such as selpercatinib are effective in RET-mutated tumors, while vandetanib and cabozantinib remain treatment options for RET-negative disease [30]. Surgery remains the preferred treatment for structurally detectable locoregional recurrence, with EBRT considered for residual microscopic (R1) or macroscopic (R2) disease, extrathyroidal extension, bulky nodal involvement, or airway compromise. Local treatments, including surgery, radiotherapy, or ablation, may also be applied to symptomatic or enlarging metastases in the bone, liver, lung, or skin [30].

In contrast, management of the PTC component follows the principles outlined in the 2015 ATA and 2025 NCCN guidelines [7,31]. Both recommend total thyroidectomy for tumors >4 cm, in the presence of nodal or distant metastases, or in other high-risk situations. The NCCN additionally includes aggressive histology, prior significant radiation exposure, family history of thyroid carcinoma, and concurrent thyroid disease as indications for total thyroidectomy. For intrathyroidal, node-negative tumors measuring 1–4 cm, lobectomy is an option; the ATA favors total thyroidectomy when postoperative radioactive iodine (RAI) or thyroglobulin (Tg) monitoring is planned, whereas the NCCN considers lobectomy sufficient when margins are negative and the opposite lobe is disease-free. The 2015 ATA guidelines recommend adjuvant RAI for patients with microscopic extrathyroidal extension or lymph node metastases; if these features are found after initial lobectomy, completion thyroidectomy may be required to enable RAI administration. In differentiated thyroid cancer, Tg remains the primary surveillance marker and should always be measured alongside TSH and anti-Tg antibodies. Elevated TSH can artificially increase Tg values, while the presence of anti-Tg antibodies may mask Tg elevation with immunometric assays. Rising anti-Tg antibody titers, even without detectable Tg, may indicate recurrence. ATA guidelines advise Tg/anti-Tg measurement twice yearly and neck ultrasound annually for all risk groups during the first two years post-treatment, with additional imaging for intermediate-and high-risk patients. TSH suppression goals vary according to recurrence risk, from 0.5–3.0 mU/L in low-risk patients with undetectable Tg, to 0.1–0.5 mU/L in intermediate-risk, and <0.1 mU/L in high-risk disease, adjusting according to the clinical course [32]. In our case, elevated Ct and Tg levels from lymph node aspirates confirmed metastatic involvement from both tumor components, prompting central and lateral neck dissection followed by RAI therapy. The decision to administer 104 mCi of 131I was made to provide adjuvant therapy for the PTC component. Contemporary guidelines recommend 1.11–3.7 GBq (30–100 mCi) for adjuvant treatment of differentiated thyroid carcinoma, while doses of 3.7–7.4 GBq (100–200 mCi) are reserved for unresectable or metastatic disease [33]. Because our patient had confirmed nodal metastases and required remnant ablation, selected 104 mCi was to maximise tumouricidal effect without much exceeding the recommended adjuvant upper limits. For RAI-refractory PTC or poorly differentiated tumours, therapies targeting specific oncogenic drivers (e.g., BRAF and MEK inhibitors) have been used. Furthermore, external-beam radiotherapy, interventional ablation and combined BRAF/MEK inhibitors may be required for locoregional metastases or RAI-refractory disease [7]. These options are generally reserved for unresectable or metastatic disease and are not indicated in our patient, who remains disease-free after surgery and adjuvant RAI.

Ultimately, the prognosis in mixed thyroid carcinoma is often dictated by the MTC component, as MTC generally shows a more aggressive course and is RAI-insensitive. In the largest available series of concurrent MTC and PTC, an Italian multicenter study of 183 patients found that most were aged ≥45 years, with 97% overall survival, 66% progression-free survival, and 45% disease-free survival beyond 10 years [34]. Despite the limitations of retrospective data and incomplete records, these findings suggest a more favorable prognosis for simultaneous MTC/DTC compared with MTC alone. Surveillance after surgery in mixed MTC/PTC cases remains particularly challenging, as standard biomarkers may behave unpredictably. While current recommendations underscore the importance of measuring thyroglobulin (Tg), calcitonin (Ctn), and CEA in tandem, the reliability of combined marker interpretation is debated, particularly when biological behavior differs between PTC and MTC elements. Marked differences in median calcitonin between series likely reflect variation in MTC component burden and histologic predominance, but findings align with published thresholds for MTC that associate rising basal calcitonin (>20–50 pg/mL) with lateral neck metastases [1,8,22].

### Strengths and Limitations

The principal strength of this case lies in its detailed clinico-pathologic and biochemical characterization of an exceptionally rare mixed medullary–papillary thyroid carcinoma. The integration of histologic findings with serum and aspirate biomarkers provides a comprehensive illustration of how multimodal assessment can help therapeutic decision-making. However, the absence of molecular genetic analysis limits insight into the clonal relationship between the two tumor components, and the relatively short follow-up period precludes definitive conclusions regarding long-term prognosis.

## 3 Conclusion

This case report highlights the diagnostic and therapeutic challenges associated with the rare occurrence of collision MTC and PTC. It also underscores that even small thyroid tumors, when composed of mixed MTC and PTC, can exhibit aggressive behavior with regional metastasis, challenging traditional biopsy guidelines that are based on tumor size. In this case, however, the patient’s surgical treatment was decided based on poorly regulated hormonal status and dysthyroid orbitopathy, and not on the tumor size, leading to a pathohistological diagnosis. Management must integrate treatment principles for both malignancies, with the overall prognosis largely determined by the MTC component. Close postoperative surveillance using calcitonin, CEA, and thyroglobulin is essential, as biomarker dynamics may be unpredictable. Nevertheless, the presence of one cancer should prompt a search for the other. Pathologists should thoroughly sample thyroidectomy specimens and use immunohistochemistry to avoid missing a second component.

## Supplementary Materials



## Data Availability

The data that support the findings of this study are available from the Corresponding Author, [Tena Šimunjak], upon reasonable request.

## Supplementary Materials

The supplementary material is available online at https://www.techscience.com/doi/10.32604/or.2025.072100/s1.
