# Supplementary material for: A Rare Collision Medullary and Papillary Thyroid Carcinoma in Autoimmune Thyroid Disease: Case Report
Source: Oncol Res. 2026 Feb 24;34(3):30. doi: 10.32604/or.2025.072100 (PMC12963683; doi:10.32604/or.2025.072100)
Supplement: Supplementary file 1 [file OncolRes-34-72100-s001.docx]

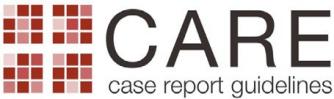

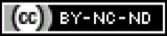

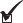
**CARE Checklist of information to include when writing a case report**

| **Topic** | **Item No** | **Checklist item description** | **Reported on Page Number/Line Number** | **Reported on Section/Paragraph** |
| --- | --- | --- | --- | --- |
| Title | 1 | The diagnosis or intervention of primary focus followed by the words “case report” | p. 1 | Title page – title |
| Key Words | 2 | 2 to 5 key words that identify diagnoses or interventions in this case report, including "case report" | p. 1 | Abstract – keywords |
| Abstract  (Structured summary) | 3a | Background: state what is known and unknown; why the case report is unique and what it adds to existing literature. | p. 2 | Abstract – Background paragraph 1 |
|  | 3b | Case Description: describe the patient’s demographic details, main symptoms, history, important clinical findings, the main diagnosis, interventions, outcomes and follow-ups. | p. 2 | Abstract – Case description (paragraph 2) |
|  | 3c | Conclusions: summarize the main take-away lesson, clinical impact and potential implications. | p. 2 | Abstract – Conclusion (paragraph 3) |
| Introduction | 4 | One or two paragraphs summarizing why this case is unique **(may include references)** | p. 3 | Introduction – paragraphs 1–2 |
| Patient Information | 5a | De-identified patient specific information | p. 3 | Case presentation – paragraph 1 (demographics) |
|  | 5b | Primary concerns and symptoms of the patient | p. 3 | Case presentation – paragraph 1 (primary concerns/symptoms |
|  | 5c | Medical, family, and psycho-social history including relevant genetic information | p. 3 | Case presentation – paragraph 1 (medical history) |
|  | 5d | Relevant past interventions with outcomes | p. 3 | Case presentation – paragraph 1 (past interventions and outcomes) |
| Clinical Findings | 6 | Describe significant physical examination (PE) and important clinical findings | p. 3-4 | Case presentation – paragraph 2 (histology) & paragraph 3 (nodal findings) |
| Timeline | 7 | Historical and current information from this episode of care organized as a timeline | p. 3-5 | Case presentation – chronological narrative (no formal timeline table) |
| Diagnostic Assessment | 8a | Diagnostic testing (such as PE, laboratory testing, imaging, surveys). | p. 3-5 | Case presentation – paragraphs 2 and 3 (histopathology, IHC, postoperative imaging and FNA) |
|  | 8b | Diagnostic challenges (such as access to testing, financial, or cultural) | p. 3-5 | Case presentation – paragraph 2 (diagnostic challenge of mixed MTC/PTC) |
|  | 8c | Diagnosis (including other diagnoses considered) | p. 3-5 | Case presentation – paragraph 2 (final diagnosis based on WHO criteria) |
|  | 8d | Prognosis (such as staging in oncology) where applicable | p. 5 | Case presentation – paragraph 3 (prognostic implication of nodal metastasis) |
| Therapeutic Intervention | 9a | Types of therapeutic intervention (such as pharmacologic, surgical, preventive, self-care) | p. 4-5 | Case presentation – paragraph 4 (total thyroidectomy and neck dissection) |
|  | 9b | Administration of therapeutic intervention (such as dosage, strength, duration) | p. 4-5 | Case presentation – paragraph 4 (extent of dissection and adjuvant RAI) |
|  | 9c | Changes in therapeutic intervention (with rationale) | p. 4-5 | Case presentation – paragraph 4 (treatment escalation based on nodal findings) |

| Follow-up and Outcomes | 10a | Clinician and patient-assessed outcomes (if available) | p. 4 | Case presentation – paragraph 5 (postoperative biochemical response and clinical status) |
| --- | --- | --- | --- | --- |
|  | 10b | Important follow-up diagnostic and other test results | p. 4 | Case presentation – paragraph 5 (follow-up imaging and tumor markers) |
|  | 10c | Intervention adherence and tolerability (How was this assessed?) | p. 4 | Case presentation – paragraph 5 (ongoing surveillance and adherence) |
|  | 10d | Adverse and unanticipated events | p. 4 | Case presentation – paragraph 5 (no adverse events reported) |
| Discussion | 11a | A scientific discussion of the strengths AND limitations associated with this case report | p. 6-13 | Discussion – paragraph 8 (strengths and limitations) |
|  | 11b | Discussion of the relevant medical literature **with references** | p. 6-17 | Discussion – paragraphs 1–7 (literature review) |
|  | 11c | The scientific rationale for any conclusions (including assessment of possible causes) | p.14 | Discussion – paragraphs 6–7 (scientific rationale) |
|  | 11d | The primary “take-away” lessons of this case report (without references) in a one paragraph conclusion | p.14 | Discussion – final paragraph (take-away message)  Conclusion paragraph |
| Patient Perspective | 12 | The patient should share their perspective in one to two paragraphs on the treatment(s) they received |  | Not applicable – patient perspective not obtained |
| Informed Consent | 13 | Did the patient give informed consent? Please provide if requested | **Yes** | **No** |

*As the checklist was provided upon initial submission, the page number/line number reported may be changed due to copyediting and may not be referable in the published version. In this case, the section/paragraph may be used as an alternative reference.
